# Cyanide Insensitive Oxidase Confers Hydrogen Sulfide and Nitric Oxide Tolerance to *Pseudomonas aeruginosa* Aerobic Respiration

**DOI:** 10.3390/antiox13030383

**Published:** 2024-03-21

**Authors:** Martina R. Nastasi, Lorenzo Caruso, Francesca Giordano, Marta Mellini, Giordano Rampioni, Alessandro Giuffrè, Elena Forte

**Affiliations:** 1 Department of Biochemical Sciences “A. Rossi Fanelli”, Sapienza University of Rome, 00185 Rome, Italy; martinaroberta.nastasi@uniroma1.it (M.R.N.); f.giordano@uniroma1.it (F.G.); 2 Department of Science, Roma Tre University, 00146 Rome, Italymarta.mellini@uniroma3.it (M.M.); giordano.rampioni@uniroma3.it (G.R.); 3 IRCCS Fondazione Santa Lucia, 00179 Rome, Italy; 4 Institute of Molecular Biology and Pathology, National Research Council, 00185 Rome, Italy

**Keywords:** hydrogen sulfide, nitric oxide, respiratory chain, *bd*-type terminal oxidases, *Pseudomonas aeruginosa*, cyanide insensitive oxidase

## Abstract

Hydrogen sulfide (H_2_S) and nitric oxide (NO) are long-known inhibitors of terminal oxidases in the respiratory chain. Yet, they exert pivotal signaling roles in physiological processes, and in several bacterial pathogens have been reported to confer resistance against oxidative stress, host immune responses, and antibiotics. *Pseudomonas aeruginosa*, an opportunistic pathogen causing life-threatening infections that are difficult to eradicate, has a highly branched respiratory chain including four terminal oxidases of the haem-copper type (*aa_3_*, *cbb_3_-1*, *cbb_3_-2*, and *bo_3_*) and one oxidase of the *bd*-type (cyanide-insensitive oxidase, CIO). As *Escherichia coli bd*-type oxidases have been shown to be H_2_S-insensitive and to readily recover their activity from NO inhibition, here we tested the effect of H_2_S and NO on CIO by performing oxygraphic measurements on membrane preparations from *P. aeruginosa* PAO1 and isogenic mutants depleted of CIO only or all other terminal oxidases except CIO. We show that O_2_ consumption by CIO is unaltered even in the presence of high levels of H_2_S, and that CIO expression is enhanced and supports bacterial growth under such stressful conditions. In addition, we report that CIO is reversibly inhibited by NO, while activity recovery after NO exhaustion is full and fast, suggesting a protective role of CIO under NO stress conditions. As *P. aeruginosa* is exposed to H_2_S and NO during infection, the tolerance of CIO towards these stressors agrees with the proposed role of CIO in *P. aeruginosa* virulence.

## 1. Introduction

Hydrogen sulfide (H_2_S) and nitric oxide (NO), similar to carbon monoxide, are endogenously produced gaseous molecules that can exhibit beneficial signalling and regulatory effects in cells and organisms at low physiological concentrations as well as toxic effects at higher concentrations [1]. As a result of this ambivalent behaviour, they play a crucial role in both physiological and pathological processes [2,3,4,5,6,7,8]. Notably, in bacteria the production of these gaseous molecules, known as “gasotransmitters”, has been suggested to be important during infection [9,10,11,12,13]. Specifically, these molecules have been reported to confer resistance to oxidative stress and antibiotics by enhancing the detoxification of reactive oxygen species caused by several antimicrobials [14] or by chemically modifying protein targets [15]. Indeed, H_2_S signalling mainly occurs via post-translational persulfidation of protein cysteine residues [16], which in turn modulates protein activity and can promote microbial virulence [17]. Proteomic profiling of *S. aureus* in response to exposure to exogenous sulfide has revealed that protein persulfidation regulates many key metabolic enzymes and genes related to virulence, influencing both cell surface composition and biofilm formation [18] Despite these beneficial roles exerted at lower concentrations, H_2_S and NO are better known for their ability to negatively affect energy metabolism and cell viability at higher concentrations, acting as inhibitors of antioxidant enzymes and respiratory oxidases by targeting Fe-S-containing and haem proteins [8]. The host immune system exploits this feature to fight infections by producing elevated levels of NO through the inducible NO synthase.

Unlike mammalians, bacteria possess branched respiratory chains with multiple electron entry sites and alternative oxidants as terminal acceptors [19]. Moreover, their flexible electron transfer pathways are reconfigured in response to changing conditions in the habitat [20], allowing certain bacteria to tolerate relatively high concentrations of NO and H_2_S. A highly branched respiratory chain is present in *Pseudomonas aeruginosa*, an important pathogen causing acute nosocomial lung, soft tissue, and systemic infections as well as chronic infections in individuals with underlying inflammatory lung diseases such as cystic fibrosis (CF) [21,22]. To date, *P. aeruginosa* chronic lung infection is the main cause of morbidity and mortality in individuals with CF. During permanence in the CF lung, *P. aeruginosa* undergoes evolutionary changes and adapted clones emerge with mutations conferring antibiotic resistance. Effective therapeutic options to treat *P. aeruginosa* infections are limited, and no antibiotic can eradicate the chronic lung infection that this bacterium establishes in CF individuals [23]. As a growing public health threat, *P. aeruginosa* is listed by the WHO as among the top priority pathogens for which new antimicrobials are urgently needed [24].

Remarkably, in recent years bacteria-specific energy metabolism has been garnering increasing attention as a therapeutic target [20]. Most antibiotics target the biosynthesis of DNA, RNA, proteins, or peptidoglycan, and are active against growing bacteria. However, these antibiotics are not effective in eradicating persistent infections where most of the bacterial cells are in slow-growing or non-growing states. Because maintenance of cellular energy and redox homeostasis is a requirement for non-growing cells, inhibition of energy metabolism is expected to be effective in killing persister cells as well [23]. 

The aerobic electron transport chain of *P. aeruginosa* includes five terminal oxidases, expressed under different growth conditions: three cytochrome *c* oxidases, *aa_3_*, *cbb_3_*-1, and *cbb_3_*-2, and two quinol oxidases, the cytochrome *bo_3_*, which belongs to the haem-copper oxidase superfamily, including all known cytochrome *c* oxidases, and cyanide insensitive oxidase (CIO), which is part of the family of copper-free *bd*-type oxidases [25]. All of these oxidases generate the proton motive force used for ATP production by catalyzing the four-electron reduction of O_2_ to 2 H_2_O. CIO is encoded by an operon containing the *cioA* and c*ioB* genes, which share high similarity with the corresponding *cydA* and *cydB* genes in *E. coli* that code for cytochrome *bd*-I [26]*. E. coli* possesses three terminal quinol oxidases: the haem-copper cytochrome *bo*_3_, and the *bd*-type cytochromes *bd*-I and *bd*-II [25]. Despite its high similarity to *E. coli* cytochrome *bd*-I, CIO is reported to have a different haem composition, in that while *bd*-type oxidases typically carry one low-spin haem (*b*_558_) and two high-spin haems (*b*_595_ and *d*), in CIO haem *d* appears to be replaced by a *b*-type haem [26,27]. As in *E. coli bd* oxidases, CIO is predicted to have high affinity for O_2_; indeed, a *cco1*/*cco2* double mutant lacking *cbb*_3_-1 and *cbb*_3_-2 was able to grow under microaerobic conditions (2% O_2_), whereas a *cco1*/*cco2*/*cio* triple mutant lacking *cbb*_3_-1, *cbb*_3_-2, and CIO did not grow under such conditions [28]. However, the affinity of *P. aeruginosa* CIO for O_2_ was later measured in a mutant strain expressing only CIO and found to be low, with a *Km* value for O_2_ of 4.0 ± 2.1 μM, significantly higher than those of *cbb_3_*-1 and *cbb_3_*-2 and comparable to those of *aa*_3_ and *bo*_3_ reported by Arai et al. [29]. These authors suggested that the inability of the *cco1*/*cco2*/*cio* triple mutant to grow under microaerobic conditions is due to the sensitivity of *bo*_3_ and *aa_3_* to cyanide, a potent respiratory inhibitor that is produced by *P. aeruginosa* under microaerobiosis and during infections as a virulence factor [30]. Given its tolerance to cyanide, CIO is indeed capable of sustaining aerobic respiration under cyanogenic conditions [31,32]. Although *P. aeruginosa* CIO can resist high concentrations (>1 mM) of cyanide, growth and survival experiments indicate that this oxidase is unnecessary for protection against endogenous cyanide under physiological conditions [32]. Consistently, the increase in *cio* gene expression in the stationary phase occurs in both the wild type and in the ∆*hcnB* mutant which is unable to produce cyanide. Intriguingly, endogenous cyanide boosts transcription of *P. aeruginosa ccoN4*, encoding an orphan *cbb*_3_ oxidase subunit, which makes the oxidase resistant to cyanide [33]. On the contrary, CIO contributes to cyanide resistance when this agent is exogenously supplied and under aerobiosis; in the paralytic model of *Caenorhabditis elegans* infection, in which cyanide is the mediating factor causing nematode death, CIO is required for full pathogenicity, suggesting a possible role for this oxidase in bacterial virulence [30]. 

A large body of evidence has demonstrated that *bd*-type oxidases, in addition to sustaining cell bioenergetics, can support virulence in several pathogens by providing protection against several stressors [25], including antibiotics [25,34,35], peroxynitrite [36,37], NO [38,39], hydrogen peroxide (H_2_O_2_) [40,41,42], reactive oxygen species produced by macrophages, and H_2_S [43]. Significant sulfate concentrations and high H_2_S production have recently been reported in CF individuals infected by *P. aeruginosa* both at the site of infection and in the expectorate [44,45]. This suggests a possible involvement of sulfide in the pathophysiology of this bacterium. 

In light of these observations, and considering that resistance of aerobic respiration to reactive nitrogen species and sulfide is likely advantageous for *P. aeruginosa* during infection, we tested the effect of sulfide and NO on CIO activity by performing respirometric experiments on membrane fractions derived from *P. aeruginosa* PAO1 and on isogenic mutants expressing either only CIO or all other oxidases except CIO as their terminal oxidases.

## 2. Materials and Methods

### 2.1. Materials

All chemicals were from Merck unless otherwise specified. Sulfide stock solutions were prepared by dissolving Na_2_S or NaHS crystals in degassed ultra-pure water (Milli-Q^®^, Merck Millipore, Burlington, MA, USA), as reported in [46]. In aqueous solution, H_2_S is in equilibrium with hydrosulfide (HS^−^) and sulfide (S^2−^) according to the pK_a1_~7.0 (H_2_S/HS) and pK_a2_~19 (HS^−^/S^2−^) measured at 25 °C. For simplicity, unless otherwise stated, the terms ‘H_2_S’ and ‘sulfide’ are employed interchangeably here to collectively indicate the H_2_S/HS^−^/S^2−^ species. The overall concentration of sulfide species in solution was quantified spectrophotometrically with DTNB according to [47], using the molar extinction coefficient ε_412_ = 14,150 M^−1^ cm^−1^ suggested by the producer. Stock solutions of NO were prepared at room temperature in a tonometer by equilibrating degassed ultra-pure water (Milli-Q^®^, Merck Millipore, Burlington, MA, USA) with pure NO gas (Air Liquide, Paris, France) at 1 atm. The concentration of NO in solution was obtained by titration with reduced beef heart cytochrome *c* oxidase according to [48]. 

### 2.2. Bacterial Strains, Growth Conditions, and Membrane Preparations

The *P. aeruginosa* PAO1 wild type strain used in this study was from the American Type Culture Collection (ATCC15692). For growth studies in the presence of sulfide, the PAO1 wild type strain and isogenic mutants with deletions in the *cioAB* locus (∆*cio*, [49,50], GeneBank accession numbers: *cio*A AAG07317.1 and *cio*B AAG07316.1) or in the *cyo*, *cco-1*, *cco-2*, and *cox* gene loci (∆*cyo*∆*cco*∆*cox*, [49]) were grown overnight in Lysogeny Broth (LB) complex medium at 37 °C with shaking (200 rpm). Following overnight growth, bacterial cells were diluted to obtain an optical density at 600 nm (OD_600_) of 0.1 in 10 mL of LB supplemented with 4 mM L-cysteine (LB-cys) in order to increase endogenous H_2_S levels [14] in the absence or presence of 200 µM NaHS as H_2_S donor. The resulting cultures were grown in 50 mL tubes at 37 °C with shaking (200 rpm). The OD_600_ of each culture was monitored every 2 h with a Lambda EZ 201 spectrophotometer (Perkin-Elmer, Waltham, MA, USA). 

*P. aeruginosa* membranes were prepared as described by Cunningham and Williams [31]. *P. aeruginosa* cultures were grown in LB medium until an OD_600_ of about 0.9 was achieved. Cells were then harvested by centrifugation at 5000× *g* and washed twice in 0.05 M phosphate buffer pH 7.0. The cell pellets were resuspended in 0.01 M phosphate buffer pH 7.0 containing 5 mM MgCl_2_, 0.1 mg/mL lysozyme, and 50 µg/mL DNAse. The cells were then lysed by sonication and debris were removed by centrifugation at 5000× *g*. The membrane fraction was collected by centrifugation for 2 h at 100,000× *g* at 4 °C and resuspended in 0.5 mL of 0.01 M phosphate buffer pH 7.0 containing 5 mM MgCl_2_. The protein content was determined by the Bradford method using Bradford reagent with bovine serum albumin as the standard.

### 2.3. Oxygraphic Measurements

Oxygraphic measurements were performed using high-resolution respirometers (Oxygraph-2k and NextGen-O2k all-in-one, Oroboros Instruments GmbH, Innsbruck, Austria) equipped with a 1.5 mL chamber and Clark-type oxygen electrodes. The assays were carried out at 25 °C in 50 mM K/phosphate buffer pH 7.0 supplemented with: (i) 0.5 mM NADH to test the activity of all terminal oxidases; (ii) 10 mM dithiothreitol (DTT) and 0.25 mM coenzyme Q_1_ to selectively reduce quinol oxidases; or (iii) 2 mM ascorbate and 0.2 mM tetramethylene-*p*-phenylenediamine (TMPD) to preferentially reduce cytochrome *c* oxidases. Where indicated, 1 mM NaCN was added as a control on the cyanide resistance of CIO. Na_2_S was used as a sulfide donor. The high-resolution respirometer was employed to perform simultaneous measurements of O_2_ and NO. To assess changes in the levels of NO in solution, an NO-selective electrode (World Precision Instruments, Sarasota, FL, USA) was used. The electrode was calibrated with subsequent additions of NO from a stock solution prepared as described previously. Oxygraphic assays were conducted in the dark, as the NO-ferrous haem adduct is light sensitive [51]. The half-maximal inhibition concentration value (apparent IC_50_) for NO, defined as the NO concentration accounting for 50% inhibition of O_2_ reductase activity of membranes, was estimated by monitoring the activity in the recovery phase from NO inhibition.

### 2.4. RNA Extraction and RT-qPCR Analyses

*P. aeruginosa* PAO1 cultures were grown overnight in LB at 37 °C with shaking (200 rpm). Following overnight growth, bacteria were placed in 50 mL tubes diluted to an OD_600_ of 0.1 in 10 mL of LB alone or supplemented with 4 mM L-cysteine (LB-cys) in the presence or absence of 200 µM NaHS. The resulting cultures were grown at 37 °C with shaking (200 rpm). Total RNA was extracted as previously described [52] from 1 mL of each culture at an OD_600_ of 1.2 (the late exponential/early stationary phase of growth). Briefly, cells were mixed with 2 mL RNA Protect Bacteria Reagent (Qiagen, Hilden, Germany)) and RNA was purified using an RNeasy Mini Kit (Qiagen), including the on-column DNase I digestion step. The isolated RNA was incubated for 1 h at 37 °C with TURBO DNase (0.2 U per mg of RNA; Ambion Austin, TX, USA) and with SUPERase-In (0.4 U per mg of RNA; Ambion). Total RNA was purified with the RNeasy Column Purification Kit (Qiagen). Three different pools of RNA were extracted for each sample in independent experiments. Purified RNA was quantified using a NanoDrop 2000 spectrophotometer (Thermo Fisher Scientific, Waltham, MA, USA). The absence of DNA was verified by PCR using the oligonucleotides FW*pqsB* 5′-CCGCTCGAGCGACCAGGGCTATCGCA-3′ and RV*pqsB* 5′-CCGGAATTCCTTATGCATGAGCTTCTCC-3′. For RT-qPCR analyses, the iScript Reverse Transcription Supermix for RT-qPCR kit (Bio-Rad Laboratories, Hercules, CA, USA) was used to synthesize cDNA from 1 µg of purified RNA. Real-time PCR was performed using iTaq Universal SYBR Green Supermix (Bio-Rad Laboratories) and the AriaMX Real Time PCR system (Agilent Technologies, Santa Clara, CA, USA). 16S ribosomal RNA was used as the internal control to normalize the RT-qPCR data in every single run and to calculate the relative FC in gene expression using the 2^−ΔΔCt^ method. *cioA*-specific primers (FW*cioA*RT 5′-GCCTCTGGCTGAAAACGAAC-3′; RV*cioA*RT 5′-GTGAGCACCTCGTAGGTCAG-3′) and 16S-specific primers (FW16SRT 5′-GAGAGTTTGATCCTGGCTCAG-3′; RV16SRT 5′-CTACGGCTACCTTGTTACGA-3′) were designed using Primer-BLAST.

## 3. Results

### 3.1. Effect of Reducing Systems on Respiration of P. aeruginosa Membranes

**Figure 1:**
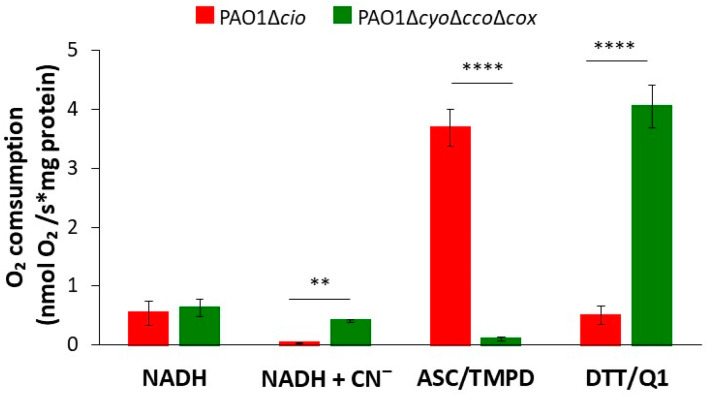
O_2_ consumption by membrane preparations from *P. aeruginosa* mutants sustained with different reducing systems. O_2_ consumption activity of membranes prepared from the Δ*cio* and Δ*cyo*Δ*cco*Δ*cox* mutants, measured at 25 °C in the presence of NADH (0.5 mM), ascorbate/TMPD (2 mM/0.2 mM), or DTT/Q_1_ (10 mM/0.25 mM). The average of at least three different experiments is reported together with the SD. For each condition, significant differences between the Δ*cio* and Δ*cyoΔccoΔcox* mutants are indicated with asterisks (**, *p* < 0.01; ****, *p* < 0.0001).

The respiratory activity of PAO1-derived mutants deleted in the *cyo*, *cco*-1, *cco*-2, and *cox* gene loci (∆*cyo*∆*cco*∆*cox*, [49]) or the *cioAB* locus (∆*cio*, [49]), respectively containing either only CIO or all other terminal oxidases except CIO*,* was initially investigated by testing the ability of membrane preparations from each strain to consume O_2_ in the presence of three different reducing systems: NADH (feeding the respiratory chain at the level of NADH-dehydrogenases, with donated electrons eventually reducing all terminal oxidases), DTT/Q_1_ (selectively reducing directly quinol oxidases), or ascorbate/TMPD (preferentially reducing directly cytochrome *c* oxidases). The results of these experiments are shown in Figure 1. When the physiological substrate NADH was used as the electron donor, the O_2_ consumption activity of both strains was similar (0.54 ± 0.21 and 0.63 ± 0.14 nmol O_2_/s*mg protein for Δ*cio* and Δ*cyo*Δ*cco*Δ*cox*, respectively). As expected, 1 mM cyanide, a potent inhibitor of haem-copper oxidases, completely suppressed NADH-mediated O_2_ consumption in the Δ*cio* strain only, indicating that CIO is the sole terminal oxidase able to sustain cyanide-insensitive respiration under the tested conditions. In the presence of ascorbate/TMPD, the Δ*cio* mutant exhibited high O_2_ consumption activity (3.7 ± 0.31 nmol O_2_/s*mg protein), while the Δ*cyo*Δ*cco*Δ*cox* mutant (with the quinol oxidizing CIO as the only terminal oxidase) displayed almost undetectable respiratory activity (0.1 ± 0.3 nmol O_2_/s*mg protein). In contrast, in the presence of DTT/Q_1_, O_2_ consumption activity by the Δ*cyo*Δ*cco*Δ*cox* mutant was much higher (4.0 ± 0.4 nmol O_2_/s*mg protein) than observed with the Δ*cio* mutant (0.5 ± 0.2 nmol O_2_/s*mg protein), indicating low expression of the *bo*_3_ quinol oxidase in the latter mutant. 

### 3.2. Effect of Sulfide on Respiration of P. aeruginosa Membrane Preparations

**Figure 2:**
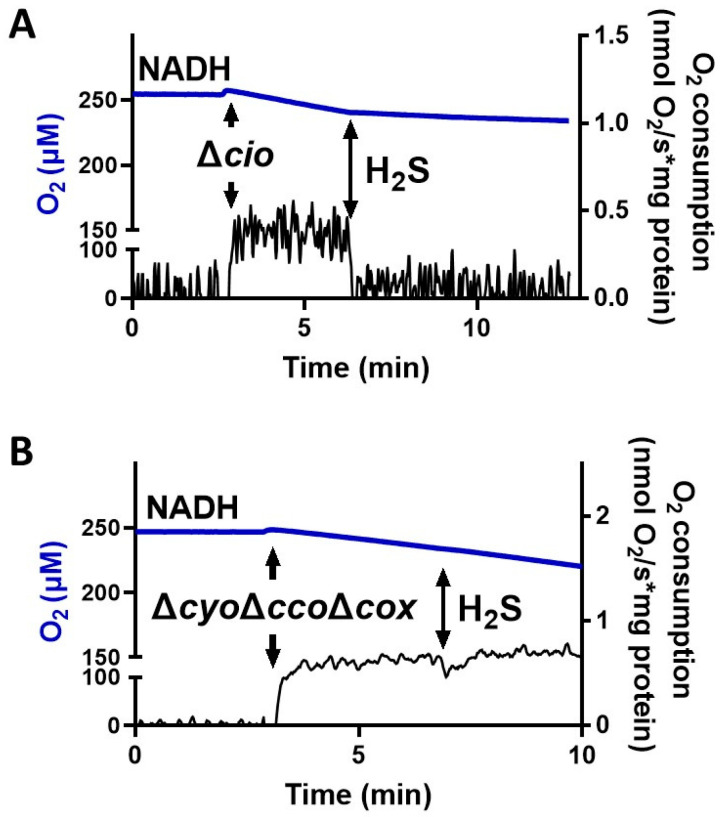
Effect of sulfide (H_2_S) on NADH-mediated O_2_ consumption activity of membranes from *P. aeruginosa* Δ*cio* and Δ*cyo*Δ*cco*Δ*cox*. Representative oxygraphic traces collected at 25 °C in the presence of 1 mM NADH before and after addition of 78 µM sulfide to membrane preparations of *Δcio* (**A**) or *ΔcyoΔccoΔcox* (**B**) mutants. Blue line: O_2_ concentration. Black line: O_2_ consumption rate. The protein concentrations of the Δ*cio* and Δ*cyo*Δ*cco*Δ*cox* membranes were equal to 0.20 and 0.11 mg/mL, respectively.

**Figure 3:**
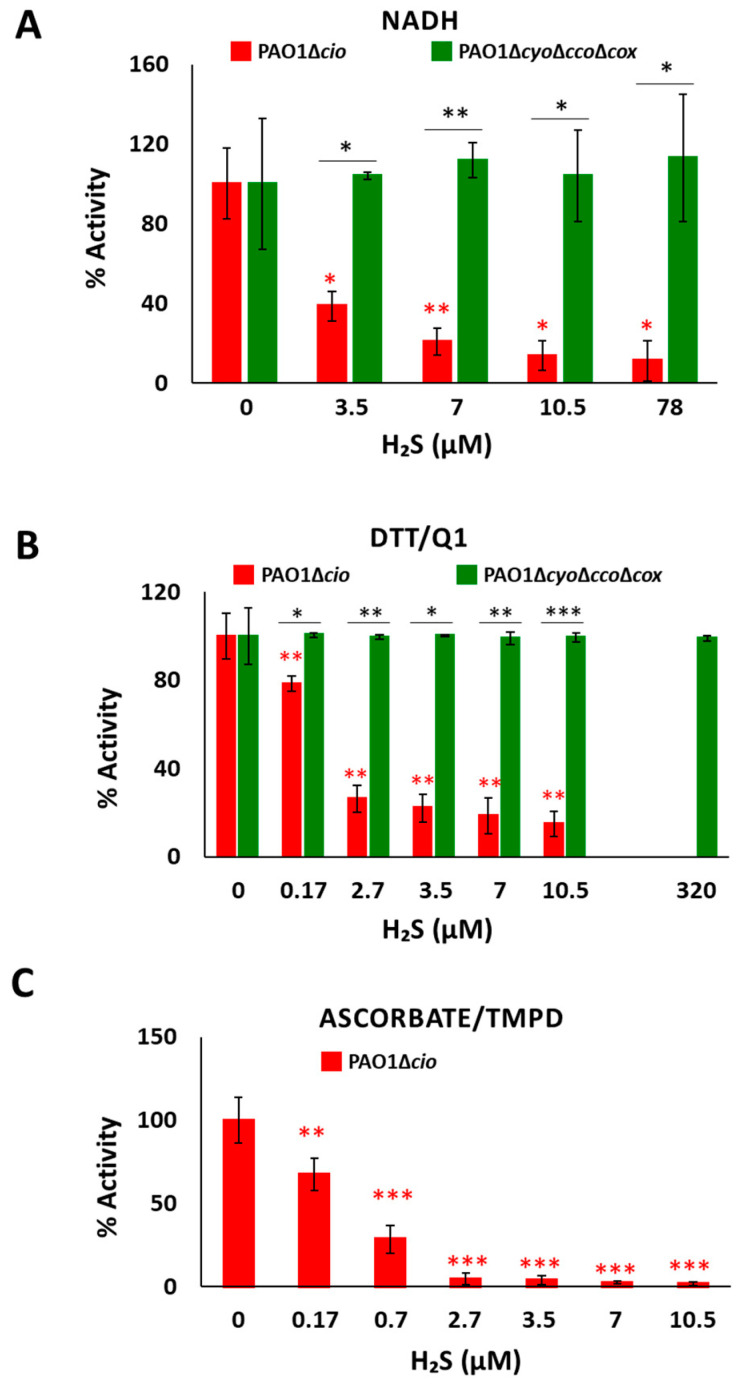
Effect of sulfide on O_2_ consumption activity of membranes from *P. aeruginosa* Δ*cio* and Δ*cyo*Δ*cco*Δ*cox*. O_2_ consumption activity of membrane preparations of the Δ*cio* and Δ*cyo*Δ*cco*Δ*cox* strains measured in the presence of (**A**) NADH (1 mM), (**B**) DTT/Q_1_ (10 mM/0.25 mM), or (**C**) ascorbate/TMPD (2 mM/0.2 mM) at increasing concentrations of sulfide. The average of three independent experiments is reported with the SD. Asterisks (*, *p* < 0.05; **, *p* < 0.01; ***, *p* < 0.001) denote significant differences between Δ*cyo*Δ*cco*Δ*cox* and ∆*cio* (panels (**A**,**B**), black asterisks) or with respect to the control not treated with sulfide (panels (**A**–**C**), red asterisks).

To test the effect of sulfide on *P. aeruginosa* CIO*,* we performed oxygraphic measurements on membrane preparations from the Δ*cyo*Δ*cco*Δ*cox* and Δ*cio* mutants. As shown in Figure 2, the addition of 78 µM sulfide completely suppressed the NADH-mediated O_2_ consumption activity of Δ*cio* (panel A) but did not affect that of the Δ*cyo*Δ*cco*Δ*cox* strain (panel B), demonstrating that CIO is insensitive to sulfide. As shown in Figure 3A, in the presence of NADH the respiratory activity of Δ*cio* mutant was drastically affected at even lower sulfide concentrations (3.5, 7, or 10.5 µM), with the O_2_ consumption activity decreasing by ca. 60% at 3.5 µM sulfide; in contrast, no change in the respiratory activity of Δ*cyo*Δ*cco*Δ*cox* membranes was observed in response to sulfide addition at all tested concentrations. To compare the sensitivity to sulfide of *P. aeruginosa* quinol oxidases*, bo_3_*, and CIO, oxygraphic experiments on the Δ*cyo*Δ*cco*Δ*cox* and Δ*cio* mutants were carried out with DTT/Q_1_ as the reducing system. Figure 3B shows that whereas CIO in the Δ*cyo*Δ*cco*Δ*cox* mutant is insensitive to up to 320 µM sulfide, the O_2_ consumption activity of *bo_3_* in the Δ*cio* mutant proved to be highly susceptible to sulfide, already being reduced by 20% at ~0.17 µM sulfide. An even higher sensitivity to sulfide was observed for cytochrome *c* oxidases in the Δ*cio* strain with ascorbate/TMPD as the reducing system (Figure 3C). 

Collectively, these results show that CIO is the only sulfide-insensitive terminal oxidase in *P. aeruginosa*, and agree with data on the model organism *E. coli* showing that the quinol haem-copper *bo*_3_ oxidase is effectively inhibited by sulfide (IC_50_ = 1.1 ± 0.1 μM) while the cytochromes *bd*-I and *bd*-II are both insensitive to sulfide [43]. As proposed for cytochrome *E. coli bd*-I, the sulfide insensitivity of CIO may arise from the lack of copper, which instead is present in the active site of haem-copper oxidases and has been proposed to be implicated in sulfide inhibition of mammalian cytochrome *c* oxidase [53]. H_2_S inhibition of mitochondrial cytochrome *c* oxidase is indeed suggested to involve the binding of the gaseous ligand at Cu_B_ in the enzyme in turnover, followed by intramolecular transfer of H_2_S to ferric haem *a*_3_ [53].

### 3.3. Effect of Sulfide on P. aeruginosa Cell Growth

**Figure 4:**
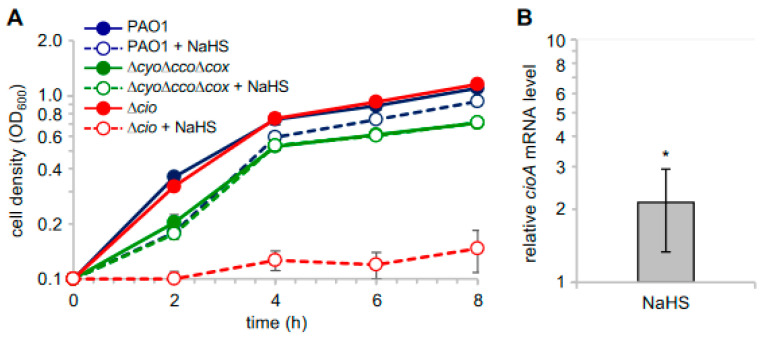
Effect of NaHS on *P. aeruginosa* cell growth. (**A**) Growth curves of the indicated strains incubated at 37 °C with shaking (200 rpm) in Lysogeny Broth supplemented with 4 mM L-cysteine (LB-cys) in the absence (solid lines, full circles; untreated samples) or presence of 200 µM NaHS (dashed lines, empty circles; treated samples). The average of three independent experiments is reported with the SD. Differences between treated and untreated cultures of both PAO1 and ∆*cio* were statistically significant after 2 h incubation (*p* < 0.001). (**B**) Fold change in *cioA* mRNA level in PAO1 grown in LB-cys supplemented with 200 µM NaHS relative to the same strain grown in LB-cys alone. The average of three independent experiments is reported with the SD (*, *p* < 0.05).

In light of the observed sulfide insensitivity of CIO, we investigated the effect of sulfide on *P. aeruginosa* cell growth by adding NaHS to cultures of *P. aeruginosa* PAO1 and its derived mutants ∆*cio* and ∆*cyo*∆*cco*∆*cox* [49]. As shown in Figure 4A, NaHS, while slightly decreasing the growth yield of PAO1, did not affect the growth of the ∆*cyo*∆*cco*∆*cox* mutant with CIO as the only terminal oxidase. Conversely, the growth of the ∆*cio* mutant was almost completely abrogated in the presence of NaHS, demonstrating that sulfide-insensitive CIO plays a pivotal role in aerobic respiration conditions in the presence of sulfide. In line with this evidence, our reverse transcriptase quantitative real time PCR (RT-qPCR) analyses revealed that *cioA* expression is upregulated by ca. two-fold in PAO1 cultures grown in the presence NaHS (Figure 4B).

### 3.4. Effect of NO on Respiration of P. aeruginosa Membranes

**Figure 5:**
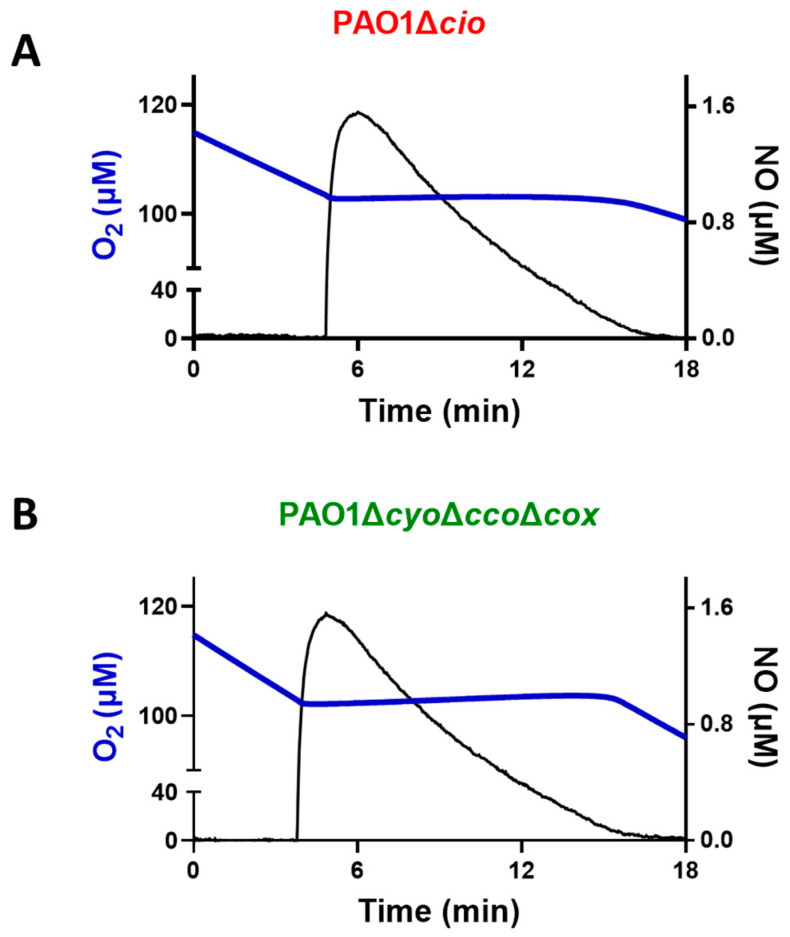
Effect of nitric oxide (NO) on NADH-mediated O_2_ consumption activity of membranes of *P. aeruginosa* mutants Δ*cio* and Δ*cyo*Δ*cco*Δ*cox.* Representative oxygraphic experiments performed at 25 °C in the presence of 1 mM NADH with membrane preparations of the Δ*cio* (**A**) and Δ*cyo*Δ*cco*Δ*cox* (**B**) strains. [NO] = 1.6 µM. The protein concentration of the Δ*cio* and Δ*cyo*Δ*cco*Δ*cox* membranes was equal to 0.27 and 0.23 mg/mL, respectively.

**Figure 6:**
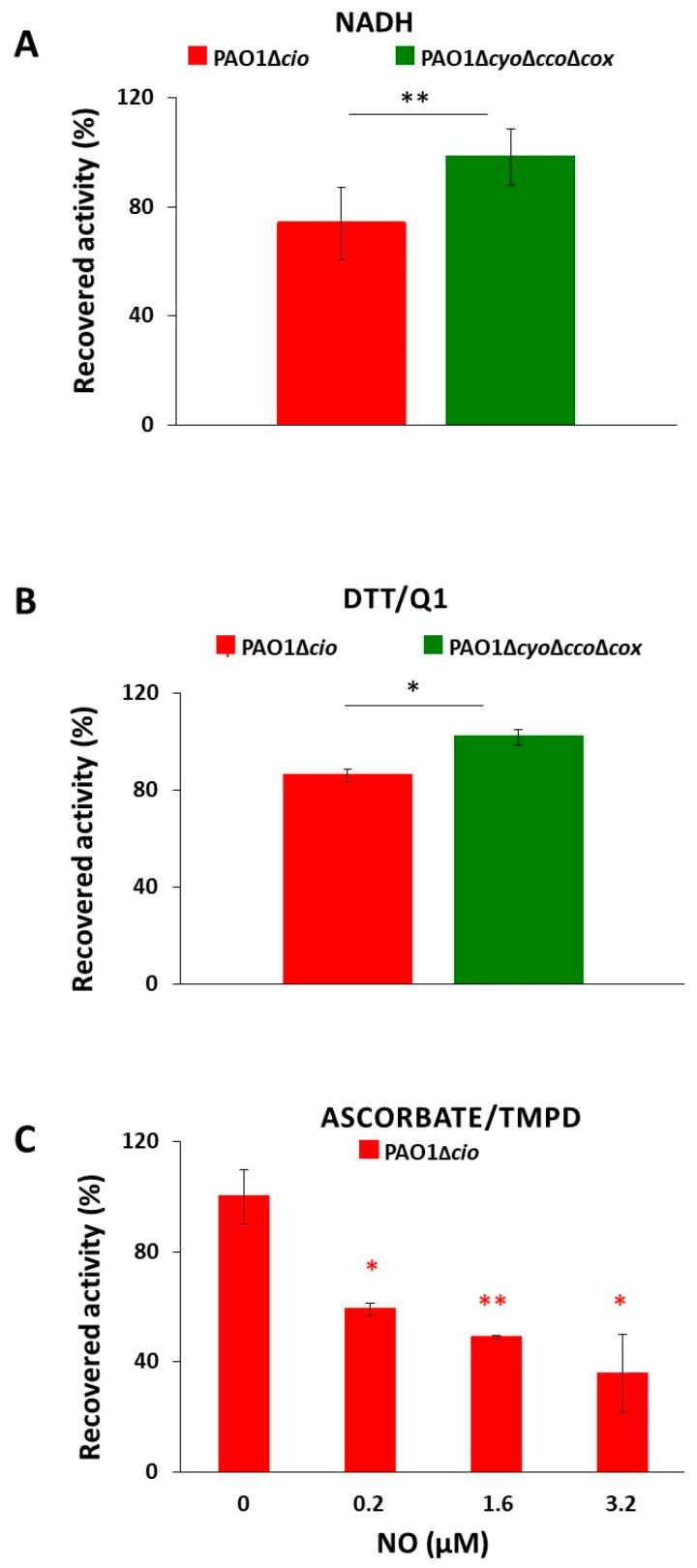
O_2_ consumption activity recovered after NO inhibition of respiration of *P. aeruginosa* Δ*cio* and Δ*cyo*Δ*cco*Δ*cox* membranes. O_2_ reductase activity recovered after NO inhibition of respiration of membranes prepared from the indicated mutants measured in the presence of (**A**) NADH (1 mM), (**B**) DTT/ Q_1_ (10 mM/0.25 mM), or (**C**) ascorbate/TMPD (2 mM/0.2 mM). The average of three independent experiments is reported with the SD. Asterisks (*, *p* < 0.05; **, *p* < 0.01) denote significant differences between Δ*cyo*Δ*cco*Δ*cox* and ∆*cio* (panels (**A**,**B**)) or with respect to the control untreated with NO (panel (**C**)).

Triggered by the finding that the NO donor sodium nitroprusside increases *cio* gene expression [54], we investigated the effect of NO on the respiratory activity of membrane fractions from both the *P. aeruginosa* Δ*cyo*Δ*cco*Δ*cox* and Δ*cio* mutant strains. Oxygraphic assays were conducted at 25 °C by simultaneously monitoring the O_2_ and NO concentrations in solution. As shown in Figure 5, in the presence of NADH as the electron donor, NO (1.6 μM) transiently and completely blocked the respiration of membrane preparations from both mutants, demonstrating inhibition by NO of all respiratory oxidases present in the membranes. When NO levels decayed upon reaction of NO with O_2_ in solution, this inhibition was relieved and respiration recovered. However, only the Δ*cyo*Δ*cco*Δ*cox* mutant regained full respiratory activity, whereas activity recovery was only partial (approximately 80% of the initial activity) in the Δ*cio* strain (Figure 5 and Figure 6A). Because similar results were obtained with DTT/Q_1_ as the reducing system, which is selective for quinol oxidases (Figure 6B), we conclude that CIO is the only quinol oxidase from *P*. *aeruginosa* to fully recover its O_2_ reductase activity after NO inhibition. Interestingly, in the presence of ascorbate/TMPD, a remarkably lower percentage of maximal respiratory recovery (50%) was observed at the same NO concentration (1.6 μM) compared to that observed with DTT/Q_1_ (cfr. Figure 6C with Figure 6B). Surprisingly, in the presence of ascorbate/TMPD, concentrations of NO as low as 0.2 μM significantly affected the ability to regain full oxidase activity (Figure 6C), implying a higher propensity of at least one cytochrome *c* oxidase to undergo NO damage compared to quinol oxidases. 

Taken together, these results show that CIO is less vulnerable to irreversible NO damage compared to other terminal oxidases in *P. aeruginosa*, possibly contributing to the resistance of *P. aeruginosa* to NO stress. 

**Figure 7:**
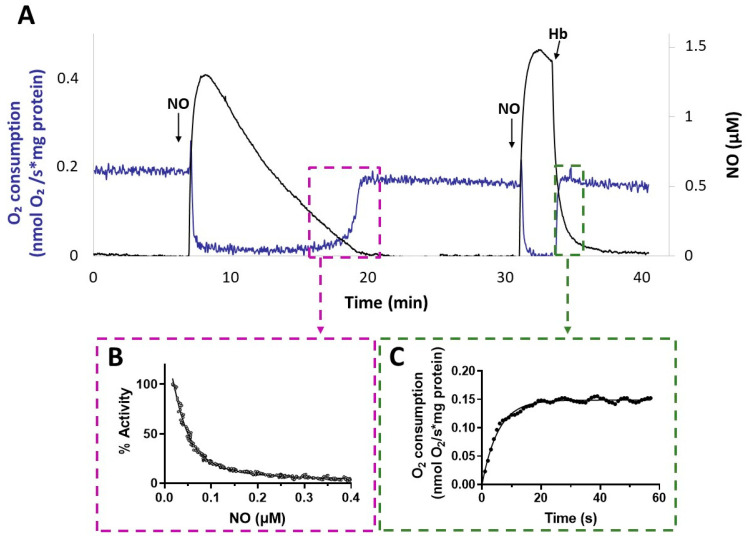
NO inhibition of CIO. (**A**) O_2_ consumption rate (blue line) and NO concentration (black line) simultaneously recorded after addition of NO to Δ*cyo*Δ*cco*Δ*cox* membranes respiring O_2_ in the presence of NADH. Following the addition of the first aliquot of NO, administered at 100 μM O_2_, respiration is transiently and fully inhibited. As NO slowly decays by reaction with O_2_ in solution, respiration recovery takes place, eventually reaching the rate observed prior to inhibition. Afterwards, addition of a second aliquot of NO at 80 μM O_2_ inhibits respiration again. Following the addition of excess HbO_2,_ which rapidly reacts with NO, fast activity recovery is observed upon NO disappearance from solution. Additions: NADH (1 mM), CIO-containing membranes (0.2 mg protein/mL), NO (1.3 µM), HbO_2_ (15 μM). (**B**) Percentage of control activity measured as NO vanishes from solution. Data from the relative boxed area depicted in panel A are plotted as a function of [NO], and data analysis yielded a half-maximal inhibitory concentration for NO value of 55 nM in this sample. The data do not intercept the y-axis, as invariably observed and tentatively explained by limitations in the NO electrode response at very low NO concentrations. (**C**) Respiration rates measured after addition of HbO_2_. Data from the relative boxed area depicted in panel (**A**) are plotted as a function of time and fitted to a single exponential (solid line), yielding an NO off-rate value of 0.18 s^−1^.

Interestingly, after NO vanished from the solution, the activity recovery was faster with membranes from the Δ*cyo*Δ*cco*Δ*cox* mutant compared to that with membranes from Δ*cio* (Figure 5). This result suggests a relatively fast dissociation of NO from the reduced haem in the CIO active site during turnover. The kinetics of activity recovery from NO inhibition of Δ*cyo*Δ*cco*Δ*cox* membranes were investigated under two distinct experimental conditions: either allowing slow decay of NO levels upon reaction of NO with O_2_ in solution, or rapidly removing the NO in solution by reaction with excess oxyhemoglobin (HbO_2_). When all unbound NO was removed rapidly from the solution with fast NO scavengers, the recovery of respiration tended to proceed at the off-rate of NO from the haem in the oxidase active site [51]. These respirometric experiments were conducted in the presence of NADH and while adding NO at an [O_2_] level of about 100 µM. As shown in Figure 7, activity recovery in the presence of HbO_2_ was remarkably faster than in its absence (panel A), proceeding at 0.18 ± 0.01 s^−1^ (panel C), which represents a lower limit value for the off-rate of NO from CIO. This value is similar to those reported for isolated *E. coli* cytochrome *bd* in the reduced state (0.133 ± 0.05 s^−1^) [55] or as integrated in the native membrane preparations of an *E. coli* mutant strain lacking the haem-copper *bo_3_* oxidase (0.163 s^–1^) [39]. The NO k_off_ values reported for *bd*-type oxidases are about 30 times higher than the ones reported for the mitochondrial cytochrome *c* oxidase and the vast majority of haem proteins.

The slower activity recovery in the absence of HbO_2_ allowed us to investigate the dependence of the respiratory activity on [NO], from which we estimated a half-maximal inhibitory concentration value for NO (apparent IC_50_) of 49 ± 18 nM NO (panel B). An apparent IC_50_ value of about 100 nM NO at 70 μM O_2_ was previously reported for the *E. coli* purified *bd*-I [38].

## 4. Discussion

Respiratory flexibility is likely to be a major contributor to the success of *P. aeruginosa* as an opportunistic pathogen. The presence of several aerobic terminal oxidases with different affinities for O_2_ may be critical for microorganisms which need to thrive in environments characterized by steep O_2_ gradients, such as *P. aeruginosa* in biofilms. However, the mechanisms regulating the expression of terminal oxidases and their function appear to extend beyond O_2_ concentrations [29,56,57,58]. The view that oxidases with low or high O_2_ affinity are respectively expressed at high or low O_2_ concentration is not always pertinent. For instance, *P. aeruginosa* expresses the high-affinity *cbb*_3_-1 during aerobic growth even under high oxygen conditions [29]; in contrast, while the low-affinity *aa*_3_ oxidase is poorly expressed at high concentrations of O_2_, its expression is upregulated in the stationary phase and more significantly under starvation of carbon, nitrogen, or iron [29]. Arai et al. suggested that the *aa*_3_ oxidase is preferentially used under low nutrient conditions, as this oxidase has the highest proton translocation efficiency of all five *P. aeruginosa* terminal oxidases and consequently the highest efficiency in generating ATP [29]. Thus, each oxidase appears to be operative under specific conditions, suggesting that modulation of the *P. aeruginosa* respiratory chain is effectively influenced by environmental conditions and stresses.

Such adaptations are important for colonization of infection sites, particularly in the lungs of CF individuals, where bacteria are challenged not only by low O_2_ levels but by the paucity of nutrients and the presence of toxic species. The low-affinity CIO is preferentially expressed during the stationary phase, when O_2_ is relatively scarce, but secondary and toxic metabolites acting as signalling molecules or virulence factors are produced [59,60]. As the amount of H_2_S significantly increases in the stationary phase [61], and as *bd*-type oxidases, at least in *E. coli*, have been shown to be insensitive to H_2_S inhibition, we hypothesized that *P. aeruginosa* CIO could be insensitive to such long-known inhibitors of O_2_ respiration as well. Consistently, we proved that CIO activity is highly insensitive to sulfide and that, remarkably, its expression is enhanced in the presence of this agent, further confirming that the expression of terminal oxidases in this pathogen is dependent on environmental stressors [43,53].

Importantly, these results strongly support the central role of CIO in *P. aeruginosa* growth and colonization potential in sulfide-rich environments, and agree with previous studies on other pathogens. Saini et al. reported that H_2_S promotes respiration and growth in *Mycobacterium tuberculosis*, *Mycobacterium smegmatis*, and *Mycobacterium bovis* BCG via cytochrome *bd*, with the other terminal oxidase cytochrome *aa*_3_ (forming a supercomplex with cytochromes *bcc*) being inhibited by host-derived H_2_S produced during infection [62]. Interestingly, endogenously produced H_2_S allows bioenergetic homeostasis by stimulating respiration, primarily via the *bd*-type terminal oxidase in multidrug-resistant and drug-susceptible clinical *M. tuberculosis* strains, but not in the non-pathogenic *M. smegmatis* [63]. While it has been known for more than 70 years that clinical strains of *P. aeruginosa* produce endogenous H_2_S [64], it was only in 2011 that a study linked H_2_S generation to *P. aeruginosa* pathogenicity [14]. In that work, the authors showed that endogenous H_2_S protects *P. aeruginosa*, as well as *Staphylococcus aureus*, *E. coli*, and *Bacillus anthracis*, from oxidative and antibiotics stress, proposing H_2_S as a general defence mechanism against antibiotic killing. However, while a number of studies have later confirmed this proposal [61,65,66], others have questioned the universality of this mechanism among bacteria [67,68], including *P. aeruginosa* [69]. H_2_S and its derived sulfane sulfur-containing species have been found to promote transcription and production of several virulence factors by activating LasR [70], which is a quorum sensing master regulator in *P. aeruginosa* PAO1. This finding points to a role of H_2_S in *P. aeruginosa* virulence and implies that CIO may be relevant during infection by allowing sulfide-resistant growth and energy production.

In accordance with the potential role of CIO in microbial pathogenicity, we report here that, under the tested conditions, CIO is more resistant to NO damage than the other *P. aeruginosa* respiratory oxidases. Experimental evidence supports that *bd*-type oxidases are key enzymes for host colonization under nitrosative stress. Deletion of cytochrome *bd*-I from a multi-drug resistant uropathogenic *E. coli* strain impaired the survival of bacteria in the mouse urinal tract [34]. The NO tolerance of this oxidase agrees with the enhanced expression of its coding genes observed under NO stress in *E. coli. S. enterica* ser. Typhimurium, *S. aureus*, *M. tuberculosis*, and *Bacillus subtilis* [25,71,72,73,74,75]. It has been proposed that the NO tolerance of *bd* oxidase is due to its ability to rapidly convert NO into nitrite during turnover and its unusually high NO dissociation rate from the active site [55]. As with most haem-copper oxidases [76,77], *E. coli* and *Azotobacter vinelandii bd*-type oxidases are rapidly, potently, and reversibly inhibited by NO through binding at reduced haem *d* [38]. Accordingly, we found that *P. aeruginosa* CIO inhibition by NO is complete and fully reversible, and that the recovery of activity is fast due to the high NO off rate [39,51,55,78].

We suggest that the fast recovery of CIO activity from NO inhibition could allow *P. aeruginosa* to cope with this potent inhibitor of aerobic respiration during infection, when the pathogen is exposed to the NO produced by eukaryotic host cells as part of the mammalian immune defence system [79,80]. To keep NO levels low and avoid its toxicity, *P. aeruginosa* is equipped with at least two efficient inducible NO detoxification mechanisms: a flavohemoglobin, which has NO dioxygenase activity under aerobic conditions and is induced by the NO donors sodium nitroprusside and *S*-nitrosoglutathione (GSNO) [81], and an NO-reductase, which removes NO under microaerobic conditions and allows intracellular survival in the NO-producing RAW 264.7 macrophage by counteracting the host’s defence systems [82,83]. Our data show that CIO may contribute to NO tolerance by allowing prompt recovery of energy production following NO stress, thereby conferring a physiological advantage to *P. aeruginosa*. Interestingly, transcription of the *cyo* gene coding for *bo_3_* oxidase is induced by GSNO [81,84,85,86,87]; however, the role of the *bo_3_* oxidase in NO resistance is unclear at present, and future studies are needed to clarify this issue. In light of the plasticity of the *P. aeruginosa* aerobic electron transfer chain and the fine-tuning of respiratory enzyme expression in response to environmental conditions, it is conceivable that other terminal oxidases are implicated in NO tolerance in addition to CIO. Indeed, as reported above, two oxidases appear to be involved in protection against cyanide toxicity under different environmental conditions [30,32].

The use of mutant strains may be a limitation of this study, as mutations can potentially influence other processes in the strain and introduce phenotypic changes. However, the growth and expression data on wild-type cells are consistent with those obtained with the mutant strains, giving us reason to believe that our conclusions are correct. Another limitation is the utilization of the PAO1 laboratory reference strain; future studies with clinical strains are needed in order to shed light on the effect of NO and H_2_S on terminal oxidase gene expression and the extent of tolerance to these gases in vivo.

## 5. Conclusions

Collectively, our data show that CIO provides *P. aeruginosa* with aerobic respiration tolerance to NO and H_2_S, noxious molecules to which this pathogen is exposed during infection, suggesting a role of CIO in *P. aeruginosa* virulence. Accordingly, we observed that exposure of *P. aeruginosa* PAO1 to H_2_S upregulates a gene coding for CIO. These new findings contribute to a better understanding of how different terminal complexes participate in the respiratory chain under various growth conditions. This study provides further evidence that high tolerance to stressors is one of the most distinctive and interesting features of *bd*-type oxidases, which have recently been recognised as attractive potential targets for the development of next-generation antimicrobials [25]. In the current scenario, where research and development on new strategies to treat *P. aeruginosa* infections has become essential, targeting bacterial energy metabolism by inhibition of respiratory chain components is a promising strategy [20].

## Data Availability

Data is contained within the article.
